# Supplementary material for: The Adaptive Alternation of Intestinal Microbiota and Regulation of Host Genes Jointly Promote Pigs to Digest Appropriate High-Fiber Diets
Source: Animals (Basel). 2024 Jul 16;14(14):2076. doi: 10.3390/ani14142076 (PMC11274041; doi:10.3390/ani14142076)
Supplement: Supplementary file 1 [file animals-14-02076-s001.zip › animals-3070719-supplementary.pdf]

Table S1 The primer sequences used in quantitative real-time PCR

Gene	Primer (5’-3’)
AQP8	F: GGTGCCATCAACAAGAAGACG
	R: CCGATAAAGAACCTGATGAGCC
CKM	F: AGAATCTGGCTGACAAGGGT
	R: GCGGAGCTCAGGGATAATGA
CNN1	F: AGCCCCACGACATCTTTGAA
	R: CCACGTTCACCTTGTTCCCT
PYY	F: AGATATGCTAATACACCGAT
	R: CCAAACCCTTCTCAGATG
SLC5A8	F: CGCAGATTCCTACTAACC
	R: GATTGTCAGTTCCACCAT
β-actin	F: TGCGGGACATCAAGGAGAAG
	R: AGTTGAAGGTGGTCTCGTGG
